# Sustainable Organic Phase Change Materials for Sustainable Energy Efficiency Solutions

**DOI:** 10.3390/polym17101343

**Published:** 2025-05-14

**Authors:** Antonella Sarcinella, Sandra Cunha, Ingried Aguiar, José Aguiar, Mariaenrica Frigione

**Affiliations:** 1 Innovation Engineering Department, University of Salento, 73100 Lecce, Italy; antonella.sarcinella@unisalento.it; 2 Centre for Territory, Environment and Construction (CTAC), Department of Civil Engineering, Campus de Azurém, University of Minho, 4800-058 Guimarães, Portugal; sandracunha@civil.uminho.pt (S.C.); id11199@vamos.uminho.pt (I.A.); aguiar@civil.uminho.pt (J.A.)

**Keywords:** sustainable organic PCMs, bio-based PCMs, waste-derived PCMs, energy efficiency, building materials

## Abstract

The growing demand for sustainable energy solutions has intensified research on phase change materials (PCMs) due to their ability to efficiently store and release thermal energy. However, traditional PCMs are often made from petroleum-derived materials or rely on processes that pose environmental concerns. The aim of this work is therefore to explore the development and use of sustainable organic PCMs, in particular those based on bio-based or waste-derived materials. Bio-based PCMs, including fatty acids, natural waxes, and biopolymers, are in fact characterized by renewability and biodegradability. Waste-derived PCMs, such as those from the lost-wax casting industry and industrial by-products, offer an environmentally friendly approach to energy storage by reusing waste materials. This paper aims to analyze the thermal, mechanical, and in-service performance of these sustainable materials, highlighting their advantages and limitations compared to the most widely used commercial PCMs. Furthermore, recent progress in the integration of sustainable PCMs into building materials is illustrated to assess their practical implementation. Challenges and limitations, as well as possible solutions and future research directions, are also discussed.

## 1. Introduction

Sustainability is a multifaceted concept that has become central to global discussions on environmental, economic, and social challenges. At its core, sustainability refers to “meeting the needs of the present without compromising the ability of future generations to meet their own needs” [1]. This broad definition includes environmental protection, economic growth, and social equity, recognizing that human activities must be conducted in ways that preserves natural resources, reduces environmental degradation, and ensures the well-being of all communities. In recent decades, sustainability has become a guiding principle for industries worldwide, leading to innovations that prioritize renewable resources, energy efficiency, waste reduction, and the minimization of ecological footprints.

When applied to materials science, sustainability focuses on the development and use of materials and processes that minimize their impact on the environment. This includes considering the entire life cycle of a material, from raw material extraction to production, use/reuse, and end-of-life disposal. In this context, sustainable materials are considered to be those obtained from renewable resources, possibly biodegradable (i.e., with zero impact at the end of their useful life), capable of reducing or offsetting environmental impacts during their production and/or use, such as carbon emissions, resource depletion, and pollution [2].

Phase change materials (PCMs) are well known as a promising technology capable of improving energy efficiency and thermal management in various applications. These materials, characterized by their ability to absorb and release thermal energy during phase transitions (from solid to liquid and from liquid to solid), offer an effective and intrinsically sustainable solution to energy storage needs. PCMs are widely used in solar energy storage devices, energy efficiency in buildings, electronic cooling technologies, pharmaceutical, healthcare, food, beverage, and even textile applications [3,4,5,6,7,8].

When used in buildings, PCMs are able to store excess heat during the day and release it at night when outdoor temperatures drop, reducing energy consumption for heating and cooling. This represents a great advantage since buildings are responsible for a significant amount of global energy consumption and CO_2_ emissions. Indeed, the integration of PCMs that store and release thermal energy can improve the energy efficiency of a building and reduce the overall environmental impact of the built environment, as widely demonstrated in the literature [9,10,11,12].

However, the production and use of common PCMs often pose significant concerns. Traditional PCMs are often associated with major environmental impacts due to their dependence on non-renewable resources: they are produced from petroleum, their production involves high energy consumption, and they are difficult to dispose of because they are mostly non-biodegradable.

More sustainable solutions are therefore being sought, in line with the objectives of reducing dependance on fossil fuels, conserving natural resources, and minimizing waste [13,14]. In the study by Cabeza et al. [15], a life cycle assessment (LCA) analysis was performed to determine whether the reduction in energy demand offered by PCMs could offset the environmental impact associated with their production (including the encapsulation process) and disposal. The study compared three different types of PCMs, namely, paraffin, salt hydrates, and esters. The results of the analysis showed that paraffin has the highest environmental impact, being 2.4 times greater than that of salt hydrates and 3 times greater than esters. Based on these findings, the study concluded that materials from renewable resources, such as salt hydrates and esters, are more environmentally friendly and should be selected to make PCMs for sustainable applications. In a similar study authored by Menoufi et al. [16], an LCA study was carried out to compare the environmental impact of esters, salt hydrates, and paraffin used as PCMs in building envelopes. The study evaluated the production, service, and disposal phases of the PCMs. The results showed that esters have a slightly lower impact on the production phase than salt hydrates. In conclusion, the use of esters and salt hydrates as PCMs led to a reduction in the overall environmental impact of approximately 10.5% and 9%, respectively, compared to paraffin.

Sustainability in the choice of using a PCM can be, then, further strengthened by selecting raw materials and production processes that are as sustainable as possible [17].

Bio-based organic PCMs represent one of the most promising sustainable options in this context. Made from renewable resources such as fatty acids, natural waxes, and biopolymers, these materials offer the added benefit of also being biodegradable. They therefore contribute to a more sustainable energy storage solution and are in line with the growing trend towards environmentally friendly technologies [18].

Natural waxes, commonly used in cosmetics and candles, have been explored for their thermal storage capabilities. Fatty acids, which are abundant in plant and animal oils, are very promising due to their latent heat storage capacities and biodegradability [19,20,21].

Even organic PCMs obtained from waste are able to improve the sustainability characteristics of this technology. The reuse of industrial by-products and/or waste generated during production processes can reduce the need for the supply of new natural resources and contribute to the reduction in industrial waste [22,23,24]. Therefore, waste-derived PCMs support a circular economy model, where the reuse of materials reduces the need for new raw materials, decreases environmental pollution, and promotes resource efficiency. This approach to the development of new PCMs also addresses the issue of waste management, a critical challenge in many industrial sectors [14].

It can therefore be stated that the use of more sustainable PCMs, whether bio-based or waste-derived, offers a double benefit that goes beyond the simple reduction in environmental impact. On one hand, these materials, derived from renewable resources or industrial waste, reduce their dependence on fossil resources and lower the energy consumption and CO_2_ emissions associated with their production compared to traditional synthetic PCMs. On the other hand, sustainable PCMs retain the ability to improve the thermal efficiency of buildings and other structures [25].

The aim of this review is, therefore, to analyze the literature related to the development and application of bio-based or waste-derived (organic) PCMs as more sustainable alternatives to traditional petroleum-derived materials. The analysis of their in-service performance will highlight the advantages and limitations of sustainable PCMs compared to traditional and commercial ones. Additionally, the integration of these PCMs into building materials will be reported, as this represents one of the most promising pathways for their practical application. This review will finally discuss the challenges that remain in the adoption of these new materials as well as future research directions needed to overcome these obstacles.

## 2. Materials and Methods

PCMs are compounds that are able to absorb and release thermal energy during their phase transitions. These materials have been, in particular, widely tested in construction materials and elements thanks to their ability to thermo-regulate the internal environment of buildings, improving their energy efficiency. PCMs help improve the thermal performance of building materials in which they are included, such as walls, floors, roof, and insulation elements [26]. Therefore, their thermal storage effect limits the need for heating/cooling devices, resulting in significant energy savings.

PCMs can be classified into different categories based on their composition, namely, organic, inorganic, and eutectic PCMs [27]. Each of these types has different thermal and physical characteristics that determine their heat storage capacity and suitability for different applications.

Organic PCMs are typically based on natural or synthetic organic compounds. These materials are characterized by a relatively high latent heat, so they can store a significant amount of energy during phase transitions and by a fairly low thermal conductivity. Common examples of organic PCMs include paraffins, fatty acids, and certain esters. Organic PCMs are often preferred for their thermal stability and ease of use, making them ideal for diverse applications, particularly in building materials and energy storage systems. However, they have some environmental disadvantages; some types of paraffin, for example, are derived from petroleum. The production of these materials can generate CO_2_ emissions, which somewhat diminishes their environmental benefits. Other paraffins, however, are derived from natural oils. There is, therefore, a growing interest in organic PCMs derived from renewable sources that can replace conventional synthetic compounds with a reduced environmental impact [28,29].

Inorganic PCMs include salts, salt hydrates, and metals. These compounds generally have a higher latent heat than organic PCMs, making them more efficient at storing thermal energy. However, inorganic PCMs are often thermally unstable, which can lead to problems during repeated thermal cycles. Salt hydrates, for example, can present problems of supercooling or deliquescence when they absorb moisture from the air. Nonetheless, inorganic PCMs are very useful in applications that require high latent heat, such as in concentrated solar power (CSP) systems or industrial processes [29,30].

Eutectic PCMs are a mixture of two or more pure PCMs, organic–organic, inorganic–inorganic or a combination of organic and inorganic. These blends are developed to overcome some of the limitations of pure organic or inorganic PCMs. Eutectic mixtures are not subject to segregation or phase separation during their solidification, unlike pure inorganic PCMs, which can suffer from phase separation. Eutectic PCMs often are able to offer a higher latent heat than organic ones while maintaining stable thermal properties. The possibility to tune the composition of eutectic PCMs makes them a very attractive option for a wide range of thermal management applications [29,31].

### Sustainable PCMs

PCMs with “sustainability” characteristics are becoming increasingly important to reduce the environmental impact of all applications in which they are used. Sustainable PCMs are those that, based on their composition or production process, contribute to reducing the environmental impact compared to conventional and commercial PCMs [13].

Bio-based PCMs are a prime example of sustainable organic phase-change materials. Among their environmental advantages, they are derived from renewable resources (i.e., not from fossil resources), are non-toxic, and are often biodegradable. Sustainable organic PCMs can also be obtained as by-products or waste from agricultural or food processing, offering an additional benefit in reducing waste. For example, some waste vegetable oils and fats from the food industry or by-products from the pharmaceutical sector can be proposed (i.e., they are effective) as PCMs. Therefore, renewable/waste-derived PCMs are particularly attractive from an environmental point of view [19,20,21].

Other materials derived from waste streams, but which are not biodegradable, offer the functional characteristics of PCMs. Such materials include industrial by-products, polymer processing waste, wax molds, waste oils, and plastic waste. Although they are not biodegradable, these organic PCMs are considered sustainable because they do not require the sourcing of new raw materials and help reduce the volume of waste that might otherwise end up in landfills, in line with the principles of a circular economy. In conclusion, even if their environmental benefits are not as direct as those of bio-based materials, they still contribute to sustainability by transforming waste into useful products that can support energy storage and thermal management in buildings [22,23,24].

## 3. Bio-Based PCMs

**Figure 1:**
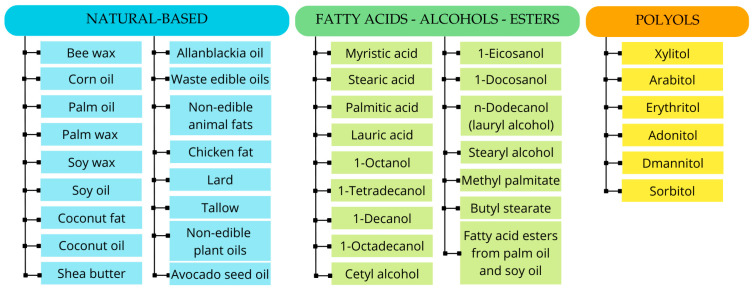
Bio-based organic PCMs organized into categories: natural-based, fatty acids–alcohols–esters, polyols.

Bio-based organic PCMs are derived from renewable sources and include substances of plant or animal origin, fatty acids, esters, and polyols [32]. Figure 1 illustrates the bio-based PCMs classified according to their nature and origin.

These PCMs can be also employed to produce eutectic mixtures, such as palmitic acid–stearic acid, myristic acid–capric acid, palmitic acid–capric acid [19], to improve the thermal properties of the original bio-based PCM.

**Figure 2:**
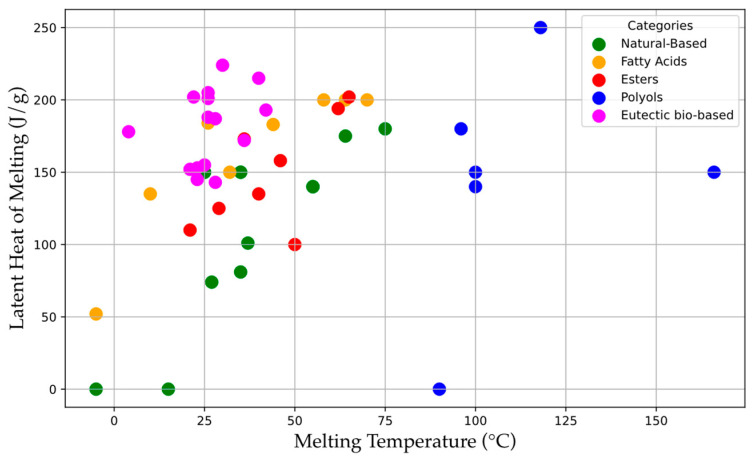
Melting temperature vs. latent heat of melting for bio-based PCMs. Source: Scopus. The data presented were derived from an analysis of publications from 2020 to 2025, conducted on 15 March 2025.

Figure 2 shows the values of melting temperatures and enthalpies for the main bio-based PCMs found in the literature.

As observed in Figure 2, most of these materials have a melting temperature between 20 and 75 °C and melting enthalpies above 100 J/g. Interestingly, eutectic mixtures composed by bio-based PCMs can offer higher melting heats, typically above 150 J/g, allowing for greater heat storage and release capacity, with a narrow melting range, from 25° to 40 °C. These features are particularly important in building applications. PCMs used in buildings must, in fact, have a melting point close to indoor comfort temperatures, i.e., between 20° and 30 °C, to efficiently store and release thermal energy during external temperature fluctuations. On the other hand, a high latent heat of melting ensures significant heat absorption without drastic temperature changes, thus improving energy efficiency, maintaining a thermal comfort inside the building.

It is worth noting that polyethylene glycol (PEG) is considered a sustainable PCM even though it is not directly derived from renewable resources (it is a synthetic polymer); for this reason, PEG has not been included in this discussion. PEG is preferred for its non-toxic nature; furthermore, despite being of synthetic origin, it is biodegradable. It has great potential as PCM due to high phase-change enthalpy, good thermal stability, and good durability under various conditions. Furthermore, the melting temperature of PEG varies depending on its molecular weight, which allows the most appropriate PEG grade to be used in applications requiring very different usage ranges [33,34].

### 3.1. Advantages and Limitations

As already highlighted, one of the main advantages of bio-based organic PCMs is their low carbon footprint. Their use is in line with the growing demand for sustainable materials that are consistent with the principles of the circular economy. Furthermore, bio-based PCMs are often quite inexpensive due to the wide availability of the raw materials from which they are derived [19,20,21].

However, as already highlighted, the use of bio-based PCMs results in several problems. In fact, they sometimes present lower thermal stability, lower heat storage capacity, and higher leakage rates during state transitions than synthetic alternatives. The melting temperature and thermal conductivity values of bio-based PCMs are not always suitable for high-performance applications, which limits their use in some sectors. Furthermore, some of them may undergo degradation under service conditions, compromising their performance over time [32].

Nevertheless, bio-based PCMs are employed in different sectors and applications. The following section illustrates, in particular, the papers in which bio-based PCMs have been proposed for construction applications.

### 3.2. Applications of Bio-Based PCMs in Buildings

The integration of bio-based PCMs into building materials is carried out using the same procedures used for conventional PCMs, namely, through direct integration (i.e., the PCM is added directly into the mortar or concrete mix) or by immersing the building material (i.e., concrete, plasterboard, porous aggregate, etc.) in a liquid PCM, which is absorbed by capillarity [19]. However, these two direct PCM inclusion procedures have some disadvantages. As an example, PCM leakage could occur, which may interfere with the cement hydration process, compromising the mechanical resistance of the building element, thus negatively affecting its lifespan.

To avoid this, PCMs can be integrated indirectly into building structures by embedding them in micro- or macrocapsules, or by employing “form-stable” methods [35], by which the PCM is absorbed into an inert support and then included in the construction element. The encapsulation method is widely recognized as one of the most effective approaches since it physically isolates the PCM inside polymeric or inorganic shells; in this way, losses during the PCM phase transitions are prevented and interactions with the building material are reduced. This method allows the preservation of the integrity of the construction element, although it reduces its mechanical resistance. On the other hand, the form-stable method is also known to be very effective, as demonstrated by the growing trend in its adoption. In fact, with this technique, it is possible to increase the thermal conductivity of the PCM, limit costs, and simplify the implementation compared to encapsulation procedures. Furthermore, the form-stable method effectively prevents PCM leakage without compromising the durability of the construction material. Finally, the use of porous and highly conductive support matrices (such as expanded graphite or silica) not only stabilizes the PCM but also manages to improve the thermal performance of the composite, making this method particularly advantageous for large-scale applications in the construction industry.

**Figure 3:**
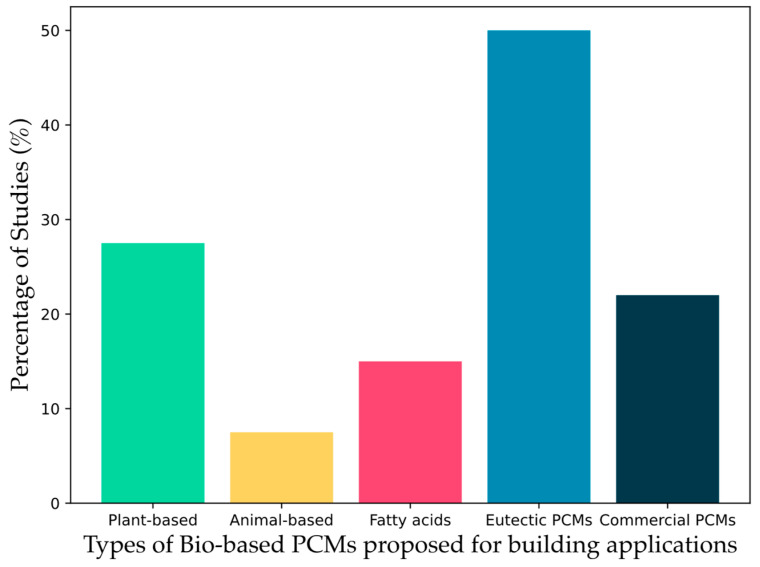
Studies on different types of bio-based PCMs proposed for building applications. (Source: Scopus. The data presented were derived from an analysis of publications from 2020 to 2025, conducted on 15 March 2025).

Figure 3 shows the results of the literature review of the paper published in the last five years on the use of bio-based PCMs in building applications. Among the studies reviewed, 22% focused on commercial bio-based PCMs; however, the exact composition of these PCMs is not always specified, leaving the nature of the bio-based materials used unclear [36,37,38,39,40,41,42,43,44]. Of the studies where the nature of the bio-based PCMs was reported, 27.5% were plant origin [45,46,47,48,49,50,51,52,53,54,55], 7.5% were animal origin [56,57,58], 15% related to single fatty acids [59,60,61,62,63,64], and 50% focused on eutectic mixtures based on bio-based components [65,66,67,68,69,70,71,72,73,74,75,76]. These data are presented in Figure 3.

**Figure 4:**
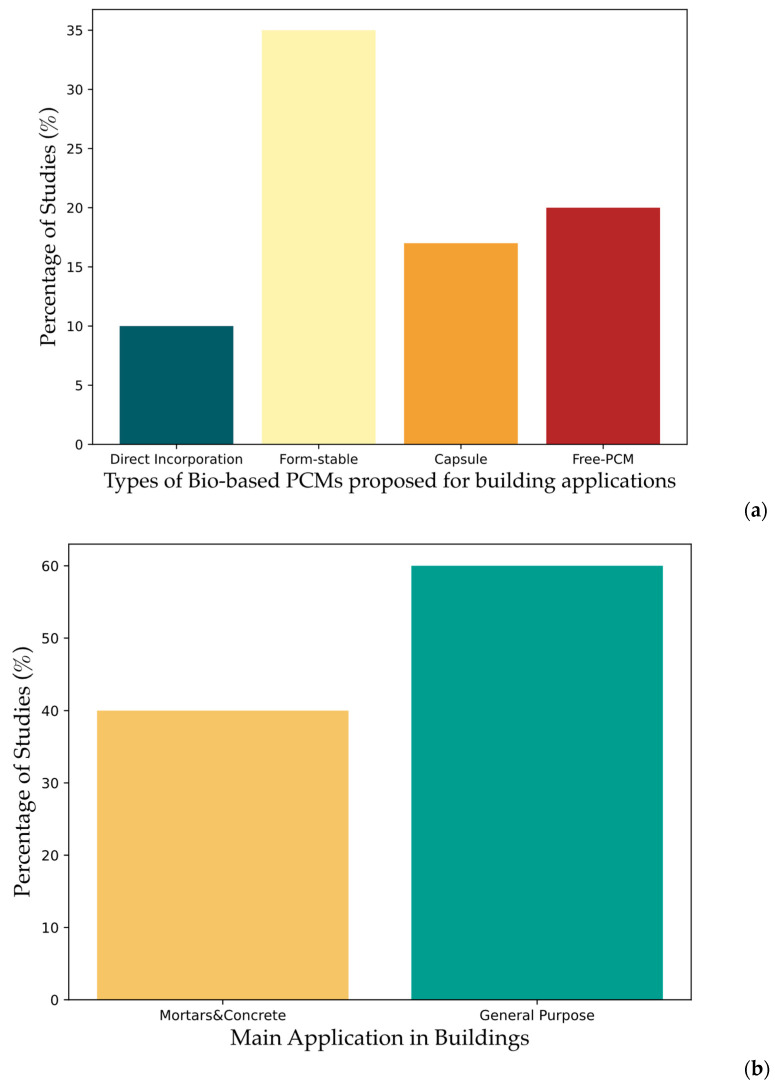
Studies on bio-based PCMs proposed for building applications, published in the last 5 years (2020–2025). (**a**) Main method used to develop bio-based PCMs for building applications; (**b**) main applications of bio-based PCMs in buildings. (Source: Scopus. Date of the analysis: 15 March 2025).

The majority of studies on eutectic mixtures, as can be seen in Figure 3, confirm the need to develop PCMs with particular thermal properties to meet specific application requirements. Regarding the methods of PCM integration in building materials, 10% of the studies reported the direct incorporation of PCMs in building materials [45,56,57,71] and 35% proposed the form-stable method, often using a natural support material in order to guarantee the sustainability of the entire system [47,49,50,51,59,61,62,63,64,65,66,67,72]. The widespread use of form-stable PCMs is justified by the fact that PCMs created using this method do not present leakage problems, as widely reported in the literature. Furthermore, this method allows the use of matrices capable of improving the thermal conductivity of the PCM (for example, using materials such as graphene, expanded graphite, SiO_2_). Finally, this solution is less complex and much less expensive than encapsulation, often allowing the reuse of waste (porous) materials as inert support. A total of 17.5% of the reviewed studies examined encapsulated PCMs, with bio-based capsules, in some cases also synthetic polymeric [48,52,53,54,60,70,74]. Finally, 20% of the papers reported the development and characterization of bio-based PCMs not integrated into any matrix or capsule (i.e., “free PCMs”). Figure 4a shows the distribution of publications describing the different methodologies used to integrate bio-based PCMs into building materials.

In terms of applications, 40% of the studies reported the inclusion of bio-based PCMs in mortars and concretes. The remaining 60% illustrated PCMs for more general purpose: in fact, no specific application in construction for them was proposed. Figure 4b summarizes these observations.

### 3.3. Thermal, Mechanical and In-Service Performance of Bio-Based PCMs

Several published papers have examined the performance of bio-based PCMs. In addition to mechanical and latent-heat thermal energy storage (LHTES) properties, their ability to limit temperature fluctuations in indoor environments is also often analyzed.

The effectiveness of bio-based PCMs in improving the thermal regulation of buildings located in high temperature regions, such as Algeria, was analyzed in [45,49,50].

Guermat et al. [45] investigated the use of a bio-based PCM made by mixing vegetable oils with purified beeswax. The PCM was found to display notable thermal properties, including an effective latent heat and a melting range that is particularly suitable for warm climates. The research also analyzed the effectiveness of the bio-based PCM when applied in buildings located in four different Algerian cities. The authors demonstrated that the integration of the PCM into building walls was able to reduce the temperature fluctuations in the internal environment by approximately 3–4 °C, particularly during the summer months, in all the cities examined. In a different study, Kehli et al. [49] developed a lightweight gypsum composite intended for hot and dry climatic regions, such as those of southern Algeria. The composite was created by incorporating in gypsum, hot water-treated barley straws and bio-based palm oil (PO) as the PCM. The use of the original PCM allowed the composite to have a high capacity to store and release thermal energy, with high thermal regulation properties. The mechanical properties were also evaluated, highlighting a slight decrease due to the reduction in density, consistent with the expected behavior of lightweight materials. However, a reduction in density is still beneficial for improving the insulation properties of the material.

Dehmous et al. [50] developed a concrete that incorporated lightweight aggregates (cLWA) impregnated with a low-cost, bio-based PCM derived from vegetable oil. Concrete containing the bio-based PCM was proposed for buildings located in warm Mediterranean climates, such as in Algeria. Three types of minerals, namely bentonite, sepiolite, and silica gel, were tested as the LWA, and two impregnation methods, namely direct impregnation and vacuum impregnation, were compared. The use of lightweight aggregates impregnated with PCM led to an average increase of 25% in the energy storage capacity of the concrete. On the other hand, the authors observed a certain decrease in both the compressive and flexural strength of the cLWA.

In a work published by Fabiani et al. [46], calorimetric analysis revealed that an expired palm oil-based PCM exhibited two distinct melting peaks (i.e., −9 °C and 24 °C), which allowed it to effectively buffer both the heat loss in the winter season and excessive temperature increases in summer. Thermal degradation analyses of palm oil indicated its stable performance in low- to medium-temperature applications. The developed PCM showed good thermal stability, maintaining its performance even after thousands of thermal cycles. The wide availability of bio-based PCMs, combined with the low cost and safety for people and the environment, makes them a sustainable choice for building applications. The low cost of materials and technology to produce these PCMs makes them accessible for large-scale use, such as in building applications.

The study conducted by Ju et al. [47] investigated the development of a novel shape-stabilized PCM (SCS@PCM) made from cenospheres (i.e., waste from coal-fired power plants), impregnated with a bio-based PCM (by-product of vegetable oil) and coated with silica coating. The composite material was integrated into cement mortars for use in building roofs. The application of a silica coating was able to limit the negative impact of SCS@PCM on the mechanical properties of mortar samples, with only a slight decrease in compressive strength compared to the reference mortar. The silica coating was also able to improve the thermal conductivity of about 13%. The developed PCM demonstrated excellent latent heat storage capabilities (melting/solidification enthalpy greater than 100 J/g) and good thermal stability, with a minimal mass loss even after extended exposure to moderate temperatures. The integration of SCS@PCM in the cement mortar led to a reduction of approximately 5 °C in the peak temperature, confirming its ability to improve thermal regulation in buildings.

A similar bio-based PCM, based on refined edible vegetable oil encapsulated in inorganic cenosphere shells sealed by silica-based coating to prevent PCM leakage, was reported in the work of Ismail et al. [48]. Then, the PCM microcapsules (SCPCMs) were incorporated into cement mortars. The 30% replacement of sand with SCPCMs resulted in a notable increase in thermal capacity compared to the control mortar. The application of a silica coating was also able to slightly improve the thermal conductivity of the mortar. On the other hand, the hydrophilic nature of the silica coating causes a decrease in the workability of the mortar, especially at higher SCPCM contents. Regarding mechanical properties, the integration of SCPCMs resulted in a slight increase in compressive strength at low concentrations, while higher concentrations (up to 30%) led to a reduction in strength. The thermal performance of the same composite PCM was analyzed in another research study [51], which showed that the integration of SPCMs resulted in reduced temperature peaks and enhanced latent heat capacity compared to uncoated PCMs. Sustainable and low-cost SCPCM materials and production process, combined with good thermal performance, provide a good basis for applications in buildings.

Sawadogo et al. [59] developed a bio-based PCM composite of hemp shives impregnated with carnauba wax (CA) for passive thermal energy storage in buildings. The hemp shives/CA composite showed promising thermal properties, with stable performance in a wide temperature range. Although the latent heat storage capacity of the composite PCM was slightly lower than that of pure carnauba wax, it remained thermally stable up to 170 °C. Concrete containing this composite PCM demonstrated an effective mitigation of indoor temperatures, with a temperature shift of approximately 5 °C and a time lag of up to 30 min during the heating and cooling phases.

**Figure 5:**
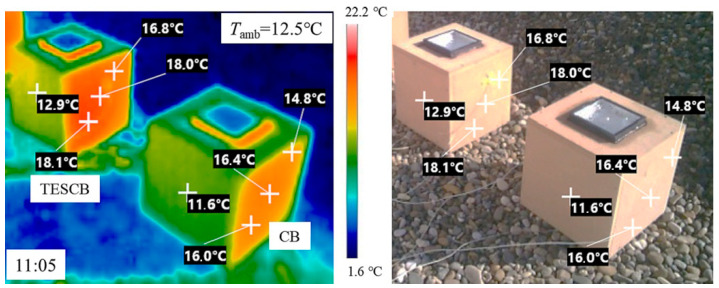
Experimental setup used on both the test and control rooms. On the (**right**), the test cells made with the reference mortar and the mortar containing the PCM; on the (**left**), the same test cells observed through the thermal camera. Reprinted from [65], with permission from Elsevier (April 2025).

The research reported in [65] aimed to develop a leak-proof bio-based PCM composite by impregnating a lauric acid–capric acid eutectic mixture (LCEM) into activated carbon from apricot kernel shells (AC-AKS). The resulting AC-AKS/PCM composite was integrated into a cement mortar containing pumice as the fine aggregate (CPM). Although the integration of PCM reduced the compressive strength of the mortar (up to 8%), the resistance values remained acceptable for the intended purpose. The mortar containing the AC-AKS/PCM composite displayed a melting temperature (around 20 °C) that is well-suited for mild thermal regulation, with latent heat that confirms a good thermal storage capacity. In Figure 5, the experimental setup employed by the authors to evaluate the in-service performance using a thermal camera of the developed PCM system is shown.

Dora et al. [61] investigated the integration of different percentages of a PCM into foam concrete. The PCM, made from exfoliated vermiculite impregnated with capric acid and ethanol (CA-AE/V), was combined with nano silica and coconut fibers. PCM-impregnated foam concrete showed a slight increase in thermal conductivity, making it effective as a heat storage medium. The melting and solidification temperatures of the PCM were in a range appropriate for building applications. Furthermore, stable thermal profiles were measured in the concrete, with reduced temperature fluctuations. The compressive strength of foam concrete was found to be suitable for making wall panels for limited PCM contents. However, at higher PCM contents, significant decreases in compressive strength were recorded.

**Figure 6:**
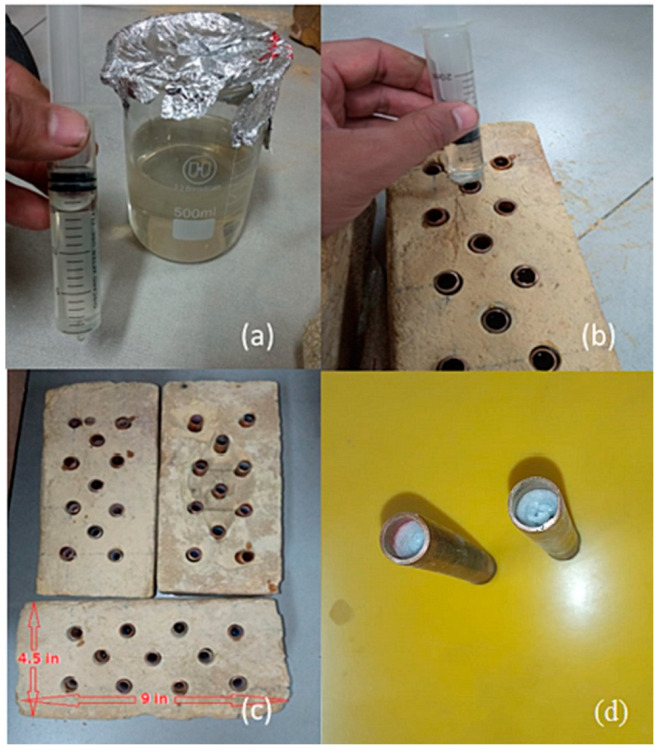
Phases of PCM integration in clay bricks: (**a**) molten PCM; (**b**) filling copper tubes with molten PCM; (**c**) copper tubes containing PCM in the bricks; (**d**) the eutectic PCM solidified inside the copper tubes. Reprinted from [71] with permission from Elsevier (April 2025).

The paper authored by Ali Taj et al. [71] reported the improvement in the thermal performance of clay bricks by incorporating a bio-based eutectic PCM. The PCM was created as a mixture of lauric acid (LA) and palmitic acid (PA), macro-encapsulated in copper tubes and then integrated into the bricks, as illustrated in Figure 6.

Analysis under real conditions showed that bricks containing the PCM exposed to direct sunlight reduced the temperature by 5 °C compared to bricks without PCM under the same exposure conditions. The inclusion of the developed PCM also led to a higher compressive strength (by approximately 23%) of the bricks compared to the unmodified bricks. Finally, the integration of PCM led to a 25–30% reduction in heat flux and a 32% decrease in thermal amplitude: the authors calculated a potential energy saving of 2.5 dollars per day thanks to the reduction in dependence on heating, ventilation, and air conditioning (HVAC) systems.

The aim of the research published by Duk Suh et al. [64] was to improve the heat storage performance of gypsum by incorporating shape-stabilized PCM. n-Heptadecane was used as the PCM and luffa fibers as the support material. n-Heptadecane was selected having a phase-change temperature range that overlaps with internal comfort temperatures (i.e., 30–40 °C). The shape-stabilized PCM integrated into gypsum showed a medium latent heat capacity. Dynamic heat transfer measurements and thermal imaging have confirmed that PCM is able to stabilize internal temperatures by reducing temperature fluctuations and increasing the time delay during thermal cycling, thus ensuring stable indoor climate and at the same time saving energy.

**Figure 7:**
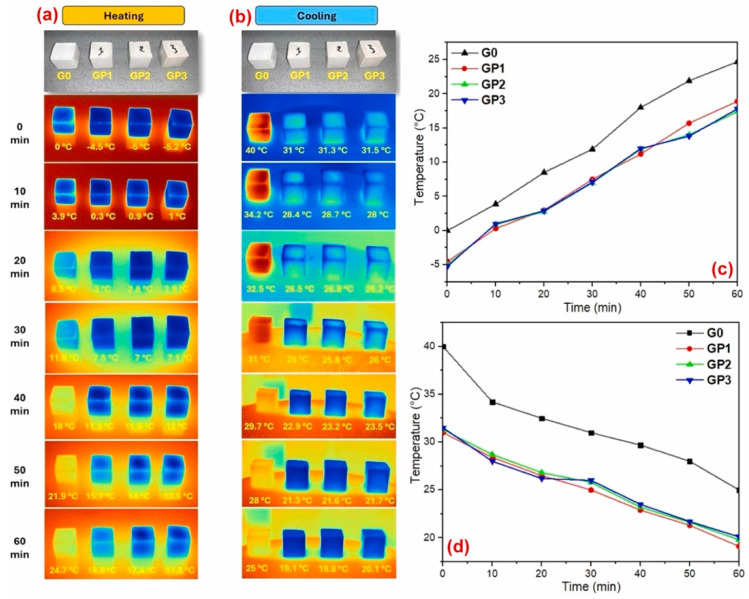
Thermal behavior of gypsum/PCM samples at different compositions: (**a**) heating phase; (**b**) cooling phase; (**c**,**d**) temperature fluctuations of gypsum/PCM. Reprinted from [60] with permission from Elsevier (April 2025).

A gypsum-based mortar including a bio-based PCM was also studied by El Majd et al. [60]. The PCM, composed of 1-Dodecanol encapsulated in biopolymers (i.e., carboxymethyl cellulose, CMC, and chitosan), exhibited excellent thermal properties, as can be seen in Figure 7.

Adding nano-Al_2_O_3_ to the PCM improved the thermal conductivity and reduced undercooling. Regarding its durability in service, the PCM showed good stability even after 100 thermal cycles. On the other hand, the compressive strength of the gypsum mortar containing PCM was significantly reduced compared to the control mortar.

Eller et al. [54] explored the use of coconut oil as a PCM to be incorporated into ceiling tiles, focusing on its potential to improve energy efficiency in tropical and subtropical climates. Simulations performed demonstrated that coconut oil is an effective PCM, particularly in climates with a high temperature range between day and night; in fact, it was able to guarantee significant energy savings, with a 32% reduction in energy needs, and a lowering of the temperature of up to 4 °C in the city of Mansa (Zambia). The widespread availability of coconut oil in tropical countries encourages its use in the construction sector, helping to support local economies.

The integration of bio-based PCMs into building materials (such as mortars, concrete) causes a decrease in mechanical properties, an effect that is well known and observed with traditional PCMs as well. However, through appropriate solutions that can be adopted for both traditional and bio-based PCMs, these reductions can be limited, allowing for the production of materials that still satisfy the performance criteria necessary for the intended use. A common approach is the use of microencapsulation or nanoencapsulation techniques; in such cases, the PCMs are enclosed within polymeric or inorganic shells [77]. This solution prevents the leakage of PCM and avoids its direct interaction with the cement matrix, thus limiting the decrease in mechanical properties. Another effective method is the adoption of form-stable or shape-stabilized systems, where the PCM is embedded within porous supporting materials [78]. Even in this case it is possible to keep the PCM confined in the matrix, limiting the losses induced by the phase change, even if the confinement effectiveness of the form stable method is lower than the encapsulation techniques. The incorporation of reinforcing fibers (i.e., of a polymer, glass, or steel nature) into the concrete can help compensate for possible losses in flexural strength [79]. It is also important to optimize the PCM content and ensure its uniform distribution within the cement mix; in fact, maintaining the PCM content below the critical thresholds (i.e., lower than 10–15% by weight) can minimize the negative effects on mechanical performance [80]. Finally, mix design modifications (e.g., reducing the water/binder ratio by adding a superplasticizer) can further help maintain the mechanical properties of mortars and concretes containing PCMs [81].

Several studies have demonstrated that bio-based PCMs exhibit excellent thermal properties, with significant latent heat capacity, which enable the effective mitigation of temperature peaks and the maintenance of a comfortable temperature inside buildings. The performance of bio-based PCMs is, therefore, comparable to that of traditional ones.

From a sustainability perspective, bio-based PCMs offer intrinsic environmental benefits as they are produced from renewable resources. It is important to underline, however, that this aspect is often overlooked in the literature. Very few LCA studies are reported in the literature to compare the environmental impact of bio-based PCMs with traditional petroleum-based PCMs [3]. Further research in this area should be conducted, to also evaluate the environmental impact of the matrix incorporating a form-stable PCM, since this material also contributes to the overall sustainability of the system. Furthermore, one of the major advantages of bio-based PCMs may be their local availability. In many cases, in fact, PCMs are applied in the same area where the materials that compose them are sourced; in this way, the need for transportation is reduced and, consequently, the carbon footprint associated with their use. This choice contributes to improving the sustainability of bio-based PCMs used in energy-efficient and environmentally friendly building solutions.

## 4. Waste-Derived PCMs

**Figure 8:**
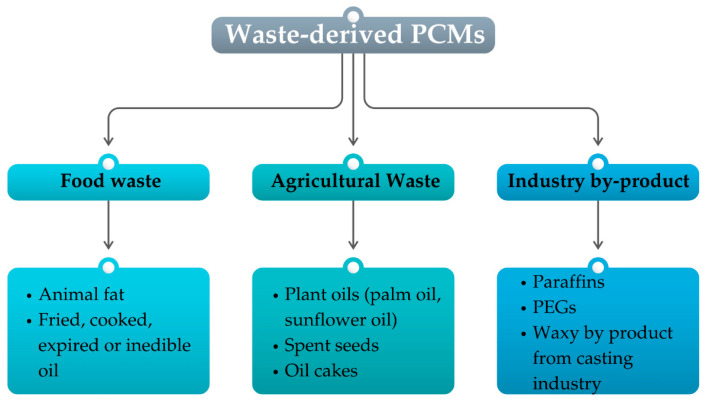
Classification of the main waste-derived PCMs.

Waste-derived PCMs are those obtained from industrial by-products or waste streams; therefore, they can offer, at the same time, solutions for waste management and thermal energy storage [23,82]. These materials can include used oils, waxes, polymers recovered from various industries, such as petrochemical and textiles, and food processing waste. The recovery of this waste to produce functional PCMs can be doubly advantageous because it would otherwise be discarded, contributing to waste accumulation and environmental pollution [13,22,24]. Figure 8 summarizes the main waste-derived PCMs.

As previously mentioned, some of the bio-based PCMs also fall under the category of waste-derived PCMs. However, they should be considered as waste materials rather than raw materials produced to act as PCMs. Waste-derived PCMs, in fact, have a higher advantage in acting as PCMs than bio-based ones from a sustainability point of view. Furthermore, many PCMs of biological origin derive from agricultural products that would no longer be available for food use. Therefore, more research efforts should be made to identify alternative solutions, such as the development of PCMs derived from food waste [19].

### 4.1. Advantages and Limitations

The use of waste-derived PCMs offers numerous benefits. They support the circular economy by transforming waste materials into materials able to store energy, helping to reduce the need for new resources. Furthermore, the economic benefit of using waste-derived materials often makes them very cost-effective, especially in sectors where waste streams are abundant and readily available. This approach can also result in significant reductions in disposal costs and environmental impacts [23,24].

However, one of the most important problems of waste-derived PCMs is the variability in the composition of the starting waste materials. Waste streams can in fact vary significantly in terms of chemical compositions and physical properties; therefore, the PCMs obtained from them can present unpredictable and uncontrollable properties, with related unsuitable performances. For example, differences in the thermal properties of waste materials can influence their phase change behavior, stability, and energy storage efficiency. To meet the standards required for industrial applications, scaling up waste-derived PCMs may require additional processes, such as removing impurities [13,23]. Industrial partnerships could certainly mitigate these problems by ensuring a stable and controlled supply of waste streams. For example, the waste supplier could invest in advanced purification technologies and collaborate with the PCM manufacturer to develop standardized procedures and certification systems. Such partnerships would improve the large-scale production of PCMs, contributing to the introduction of waste-derived PCMs into commercial markets.

Despite these problems, several examples of waste-derived PCMs are reported in the literature. For example, waste oils from food processing or the petrochemical industry have been reused to make PCMs; plastic waste has also been proposed as a material for thermal energy storage in buildings [20].

For a better understanding of the main characteristics and applicability of the two main categories of sustainable PCMs, Table 1 summarizes the main advantages and disadvantages of bio-based and waste-derived PCMs. The environmental, economic, and technical factors that influence their use provide a clear comprehension of their strengths and limitations in different contexts.

### 4.2. Applications of Waste-Derived PCMs in Building

The literature on waste-derived PCMs is often misleading, as many studies refer to form-stable PCMs based on matrices that are derived from waste rather than to PCMs actually obtained from waste. The paucity of experimental work on waste-derived PCMs makes it difficult to draw definitive conclusions about their capabilities and performance. However, the limited number of studies focusing on waste-derived PCMs show good results, suggesting that further research on these materials is needed [83,84,85,86,87,88].

### 4.3. Thermal, Mechanical and In-Service Performance of Waste-Derived PCMs

In a work conducted by Irsyad et al. [83], waste cooking coconut oil (WCCO) was found to offer good thermal energy storage capabilities. The study revealed that repeated frying alters the fatty acid composition of the oil, with a significant increase in methyl arachidate and methyl heptadecanoate. The thermal properties of WCCO, i.e., a good heat capacity during the melting phase, indicate that similarly to virgin coconut oil (CCO), it could be suitable for thermal energy storage applications. However, this material has some limitations, namely, low thermal conductivity, a wide range of phase change temperatures, and undercooling, all characteristics that could affect its efficiency in practical applications. Nonetheless, WCCO represents an interesting solution to create sustainable PCMs if its characteristics were appropriately optimized.

Boussaba et al. [84] studied a waste bio-based material to produce solar energy storage systems in buildings, particularly in hot and dry climates. The waste PCM, composed of a mixture of different vegetable fats from the food industry, showed a melting temperature of about 35 °C and a significant latent heat capacity. It was, therefore, proposed as a passive long-term thermal energy storage (LHTES) system for buildings characterized by large temperature variations. The results of the study suggested that the material is capable of improving indoor thermal comfort, in particular by mitigating temperature variations during summer heat waves if included in non-structural elements.

**Figure 9:**
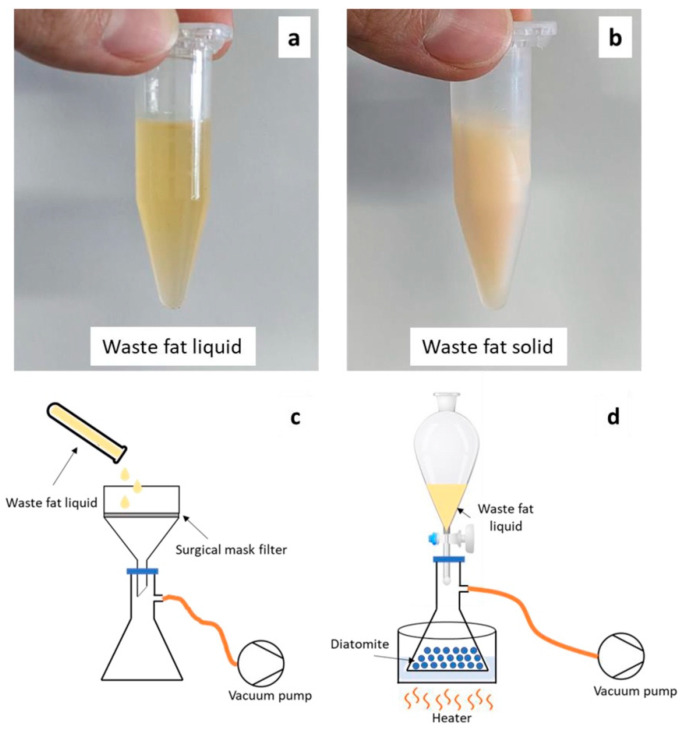
(**a**) Waste fat in liquid form and (**b**) waste fat in solid form used during the impregnation procedure for (**c**) surgical mask filter and (**d**) diatomite. Reprinted from [85], with permission from Elsevier (April 2025).

Bragaglia et al. [85] studied the use of waste fat from the pork sausage cooking process to produce waste PCMs. The waste fat, composed of both saturated and unsaturated fatty acids, presented a melting temperature of 32 °C, i.e., still suitable for indoor applications. The waste fat was included in two different matrices, namely, biosilica (diatomite) and polypropylene (PP) non-woven mats, this latter was obtained from surgical mask filter waste. The method developed to produce the two waste PCMs is illustrated in Figure 9.

Both matrices were able to effectively encapsulate a high amount of waste PCM, while maintaining unchanged thermal properties. A simplified analytical model has been developed to simulate the thermal performance of a building wall incorporating the developed PCM: a 37% reduction in thermal power was calculated compared to the wall without PCM. These results confirmed that waste cooking fat can represent an effective and eco-sustainable solution for passive heating/cooling applications in buildings.

In an experimental study performed by Rashid et al. [86], the performance of different waste materials and industrial by-products as potential PCMs for buildings was evaluated. These materials were inserted into a sandwich made up of layers of geopolymer concrete. The reference was a geopolymer concrete block. The materials tested included expanded polystyrene foam, polyurethane foam, rubber tires from industrial waste, date palm from agricultural waste, and two type of paraffin-based PCMs (PCM-30 and PCM-42), obtained as by-products from the petrochemical industry. With an appropriate choice of waste materials, a 47% reduction in cooling requirements in hot climates and a 59% reduction in heating requirements in cold climates have been measured.

An investigation conducted by Sarcinella et al. [87] explored the possibility of exploiting by-products from the lost wax casting industry as low-cost organic PCMs. With the two PCMs obtained as by-products from the lost wax casting industry, two commercial PCMs, namely paraffin and microencapsulated PCMs, were analyzed for comparison purposes. The results showed that the waxy by-products from the casting industry offered stability under repeated (100) thermal cycles comparable, and in some cases superior, to commercial PCMs. In fact, after 100 thermal cycles, all PCMs maintained their chemical and thermal properties, ensuring excellent energy storage capacity in service. Furthermore, while commercial PCMs were more sensitive to high temperatures, the waxy by-product showed higher thermal resistance.

From an LCA perspective, only a few of the previous studies has measured the impact of these materials on the environment; their ability to improve thermal efficiency in the buildings in which they are applied is mainly demonstrated. On the other hand, considering that traditional PCMs are derived from petroleum and require a lot of energy during their production, waste-derived PCMs utilize industrial and agricultural by-products, thus helping to reduce environmental impacts. A life cycle analysis of fatty acid-based PCMs from used cooking oil has shown a 40% reduction in greenhouse gas (GHG) emissions compared to the production of paraffin-based PCMs [46]. This results in a saving of approximately 2.5 kg of CO_2_ equivalent for every kilogram of PCM produced. The sustainability of PCMs obtained as agricultural by-products, such as soybean oil waste, is demonstrated by a 35% reduction in energy requirements during their production, with a saving of approximately 1.2 MJ/kg. Finally, waste-based PCMs (i.e., biomass) used in solar energy systems can offset 15 tons of CO_2_ per year for a 50 kW solar thermal system [88].

## 5. Conclusions

The use of phase change materials, PCMs, is an effective and sustainable way of ensuring the thermal comfort of buildings. In fact, the integration of PCMs in construction materials allows a reduction in the use of fossil resources for indoor heating/cooling, at the same time allowing a significant reduction in CO_2_ emissions. If organic PCMs are developed from sustainable raw materials, such as bio-based materials or industrial/agricultural waste, the environmental benefit will be twofold. Each of the different typologies analyzed presents advantages, not only from an environmental point of view, but also applicative disadvantages. As an example, bio-based PCMs are cost-effective but have a wide melting temperature range and not very high melting enthalpies, values that are not always adequate for all practical applications. They may also present thermal stability problems and leakage phenomena during thermal cycles. The form-stable method by which these PCMs are embedded in building materials can solve the leakage phenomena, also allowing the exploitation of porous waste materials capable of absorbing the PCM. Furthermore, the development of eutectics obtained from bio-based PCM mixtures allows systems to be obtained with very low melting ranges and higher melting heats.

On the other hand, there is a growing interest in using waste materials and industrial by-products to produce organic PCMs capable of storing thermal energy in buildings. The use of waste materials/industrial by-products, often easily available in large quantities and at limited costs, in materials capable of storing energy also benefits the environment from other points of view; it limits the need for new resources, reducing the entry of waste into the environment, and minimizing the related disposal costs. However, considering the variability in composition and characteristics of waste, further research is needed to optimize the properties of these waste PCMs, improve their durability, and ensure their scalability in large-scale applications. Finally, future research should address the following aspects: (1) comprehensive life-cycle cost analysis to highlight the potential economic advantages of sustainable PCMs compared to traditional ones; (2) the development of bio-based eutectic mixtures with tailored thermal properties; (3) the optimization of the stability and long-term performance of waste-derived PCMs; (4) the development of new specific standards to evaluate the performance, durability, and environmental impact of bio-based and waste-derived PCMs when integrated into building materials. It would be beneficial, and not only for the environment, to achieve an interdisciplinary integration between materials science and building design to promote the field implementation of these sustainable solutions.

## Figures and Tables

**Table 1:** Summary of the main advantages and disadvantages of bio-based and waste-derived PCMs.

Type of PCMs	Advantages	Disadvantages
**Bio-based PCMs**	▪ Low carbon footprint. ▪ Support circular economy. ▪ Inexpensive due to abundant raw materials. ▪ Widely used across different sectors.	▪ Lower thermal stability. ▪ Lower heat storage capacity. ▪ Higher leakage rates during phase change. ▪ Melting temperature and thermal conductivity not always suitable for high-performance uses. ▪ Risk of degradation over time.
**Waste-derived PCMs**	▪ Support circular economy. ▪ Highly cost-effective. where waste streams are abundant. ▪ Reduction in disposal costs and environmental impact.	▪ High variability in composition and properties. ▪ Unpredictable phase change behavior and stability. ▪ May require additional purification to meet industrial standards. ▪ Scalability depends on supply chain.
